# Imaging classification and BIRADS assessment of cystic breast lesions with pathologic correlates. a 5-year experience in Zaria, North West Nigeria

**DOI:** 10.4314/ahs.v23i3.31

**Published:** 2023-09

**Authors:** Sefiya Adebanke Olarinoye-Akorede, Suleiman Lawal, Mohammed Zaria Ibrahim

**Affiliations:** Department of Radiology, Ahmadu Bello University Teaching Hospital, Zaria Nigeria

**Keywords:** Breasts, Cysts, BIRADS, ultrasound, pathology

## Abstract

**Background:**

Breast cysts encompass a variety of pathologies, both benign and malignant. Therefore, classifying cysts into different categories is needful to develop a management algorithm. This study aimed to describe and distinguish between simple, complicated and complex cysts; and compare the final BIRADS assessment with pathologic findings.

**Materials and methods:**

A 5-year retrospective review of our ultrasound database identified two hundred and seventy patients with cystic breast lesions. They were divided into simple, complicated and complex cysts according to ultrasound characteristics based on shape, orientation, margin, wall thickness, internal features (echogenicity, septa, mass) posterior acoustic features, surrounding tissue vascularity. The final BIRADS assessment was correlated with histological findings.

**Results:**

There were two hundred and sixty-six (98.5%) females and four (1.5%) males with a mean age 34.9 ± 11.8 years. The commonest presentation was a palpable mass, in 70% of the patients. There were 89 (33.0%) simple cysts, 61 (22.6%) complicated cysts and 120 (44.4%) complex cysts.

**Conclusion:**

Majority of the breast cysts (83%) were benign with overall 17% incidence of malignancy. Complex cysts were the most frequent cyst type in our study, it is also the category most frequently associated with breast cancer, obviating the need for histology.

## Introduction

Breast cysts are a common finding in ultrasound imaging, especially in pre-menopausal women. The patient may complain of a breast mass, pain, nipple discharge or the patient may be asymptomatic in which case, the cyst is discovered at routine examination or during breast evaluation for a different complaint. Since the nature of a breast mass cannot be adequately discernible clinically, imaging is imperative.

Ultrasound (USS) exceeds mammography and clinical palpation in distinguishing a solid lesion from a cyst.^1^ With modern techniques like doppler interrogation, tissue harmonics and spatial compounding techniques 2,3, there is enhancement of lesion detail which allows further classification of cystic breast lesions into simple, complicated and complex cysts, thus guiding clinical decision making. USS also allows for image guided intervention like percutaneous drainage, USS-guided biopsy and therapeutic cyst aspiration.

Simple cysts result from dilatation and effacement of the terminal duct lobular unit. According to the sonographic criteria described by Stavros 4, 5, a simple cyst is uniformly anechoic, well-circumscribed wall with a thin echogenic capsule, increased through-transmission, and thin edge shadows. There should be no intra-cystic material or mass. When a simple cyst contains internal echoes either from pus, blood or cells, it becomes a complicated cyst.^6^,^7^ However, where a solid (intramural) component is present within a cyst, it is termed a complex cyst. 6-9 Generally, simple cysts and complicated cysts are classified as benign. Because complex cysts may be benign or malignant, histologic confirmation is required. 7,8

## Materials and Method

Our study is a retrospective review of our out-patient records, radiology and pathology data bases from January 2016 to January 2020 following ethical approval from our institution.

From these, we identified the sonograms of 270 patients that were diagnosed with cystic lesions. The ultrasound scan was done using a Mindray DC-8 (China) with a transducer frequency of 7-12MHz. From the sonographic examinations, we recorded the lesion number, breast side, shape, orientation, margin, wall thickness, internal features (echogenicity, septa, mass or solid component), posterior acoustic features, surrounding tissue vascularity. The cysts were then classified as simple, complicated or complex cyst, 4,5 as follows:

A simple cyst is uniformly anechoic, well circumscribed with a thin echogenic capsule (wall thickness < 0.5mm) and increased through-transmission.

Complicated cysts included those cysts that met all the criteria for simple cysts but in addition contained low level internal echoes or thin internal septa of less than 0.5mm thickness.

A cyst was recorded as complex if it had a wall or septal thickness greater than 0.5mm, if there was the presence of a mural nodule or if it contained a solid mass. In addition, we included a solid mass with cystic component as complex cyst.

Using the ACR guidelines for breast ultrasound,^10^ simple cysts were assigned with BI-RADS 2(benign); complicated cysts BI-RADS 3(probably benign) while complex cysts BI-RADS 4 or 5.

Imaging, cytology, histopathology results and clinical response were all reviewed to establish the final diagnosis.

## Results

There were a total number of two hundred and seventy patients (mean age of 34.9 11±.8 years; range 4 -74 years. They consisted of 266 (98.5%) females and 4 (1.5%) males.

The highest incidence of breast cysts was among 20-29 (32.6%) and 30-39 (30%) years age group. Figure 1 Out of the 266 female patients, 234 (88.0%) were pre-menopausal while 32 (12.0 %) were post- menopausal. There was no history of exogenous hormone therapy in any of the patients.

**Figure:**
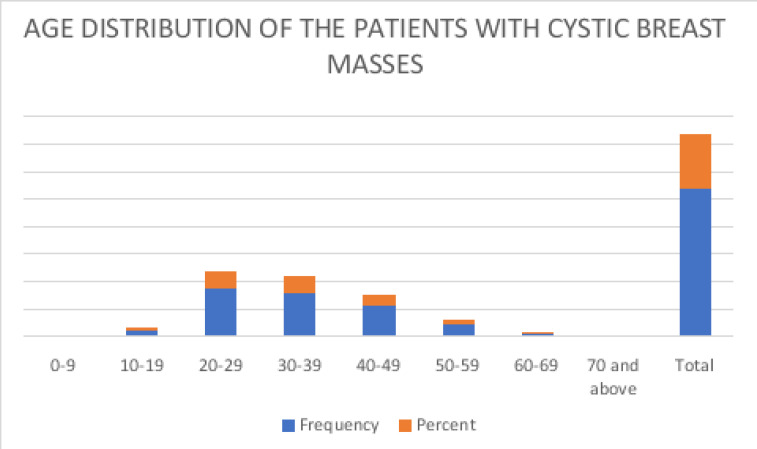
Figure 1

The symptoms evaluation was: Asymptomatic 7(2.6%), palpable mass 189 (70 %), breast pain 51(18.9%), nipple discharge 8 (3.0%), mass and pain 15 (5.6%). Table 1 Regarding the description of the cysts (Table 2) they were solitary cysts in 186 (68.9%) patients but multiple in 84 (31.1 %) patients. Right sided lesions were seen in 161 (59.6%) patients; left sided in 93(34.4%) and bilateral lesions in 16 (5.9%). There were simple cysts in 89 (33.0%), complicated cysts in 61 (22.6%), and complex cysts in 120 (44.4%) of the patients.

**Table 1:** Presenting complaints of patients with breast cysts

Complaints	Frequency (n)	Percent (%)
Asymptomatic	7	2.6
Pain	51	18.9
Mass	189	70.0
Nipple discharge	8	3.0
Mass and pain	15	5.6
Total	270	100.0

**Table 2:** Description of breast cysts

Number of cysts	Frequency	Percent
Single	186	68.9
Two	61	22.6
>two	23	8.5
Total	270	100.0
**Lesion side**		
Right breast	161	59.6
Left breast	93	34.4
Both breasts	16	5.9
Total	270	100.0
**Cyst classification**		
Simple cyst	89	33.0
Complicated cyst	61	22.6
Complex cyst	120	44.4
Total	270	100.0

According to the ACR BIRADS classification,10 the commonest category was BIRADS 3 (probably benign) 96/270 (35.6%), closely followed by BIRADS 2(benign) which accounted for 92/270 (34.1%) patients. Overall, benign category (BIRADS 2 &3) accounted for 188(69.7%) of our cases. The malignant category on USS accounted for 82 (30.3%) of cases; with BIRADS 4(Suspicious) being 51 (18.9%), BIRADS 5 (Highly suggestive for malignancy) being 29(10.7%) and BIRADS 6 (Biopsy proven malignancy) being 2 (0.7%) respectively. Table 3 Also, when compared with final histologic diagnosis, the overwhelming majority of the breast cysts were benign in 224/270 (83%), with an overall incidence of malignancy of 46/270 (17.0%). Table 3

**Table 3:** Final BIRADS category versus histology

BIRADS Category	Benign Histology	Malignant Histology	Total N (%)
2	90	2	92 (34.1)
3	93	3	96 (35.6
4	33	18	51 (18.9)
5	7	22	29 (10.7)
6	1	1	2 (0.7)
**Total**	224	46	270

In comparing the cyst classification with histology, we found out that among the 89 cases with simple cysts,98.9% were benign and 1.1% turned out histologically malignant; among the 61 patients with complicated cysts, 96.7% were benign and 3.3% cases were malignant while among the 120 complex cysts, 64.2% were benign and 35.8% were malignant. Table 4

**Table 4:** Cyst classification versus histology

Cyst classification	Benign (%)	Malignant (%)	Total
Simple cyst	88 (98.9)	1 (1.1)	89
Complicated cyst	59(96.7%)	2 (3.3)	61
Complex cyst	77(64.2)	43(35.8)	120

**Figure 2:**
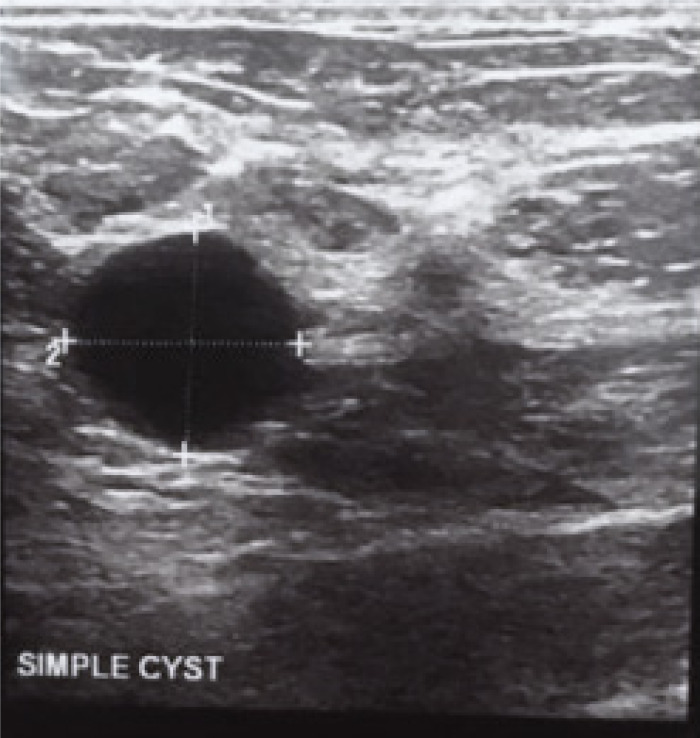
Simple cyst A 26-year-old female who complained of a painless lump in her right breast Ultrasound scan showing an anechoic mass with thin perceptible wall, through transmission and posterior acoustic enhancement Diagnosis: simple cyst

**Figure 3:**
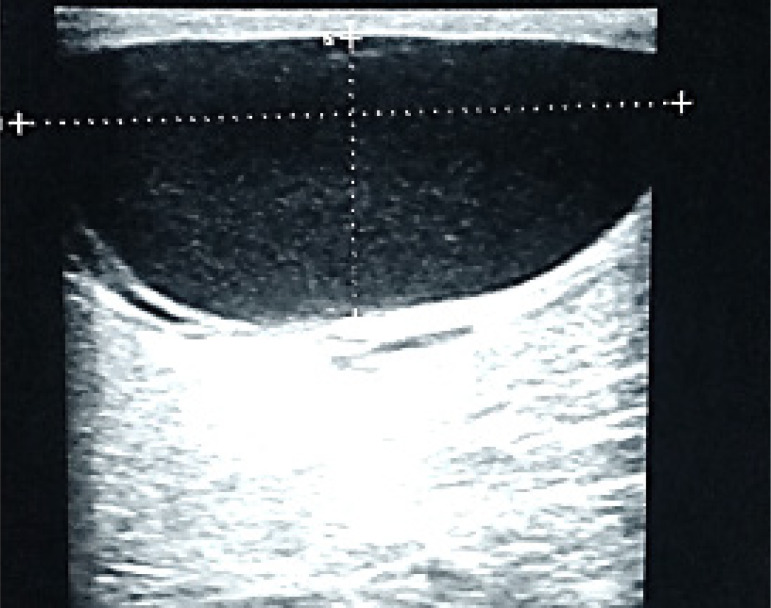
Complicated cyst A 35-year-old lactating female who complained of a non-tender firm mass in her left breast Ultrasound scan showing an oval shaped anechoic mass with moving, homogenous, echogenic internal material. Needle aspiration yielded milk Diagnosis: Galactocele

**Figure 4:**
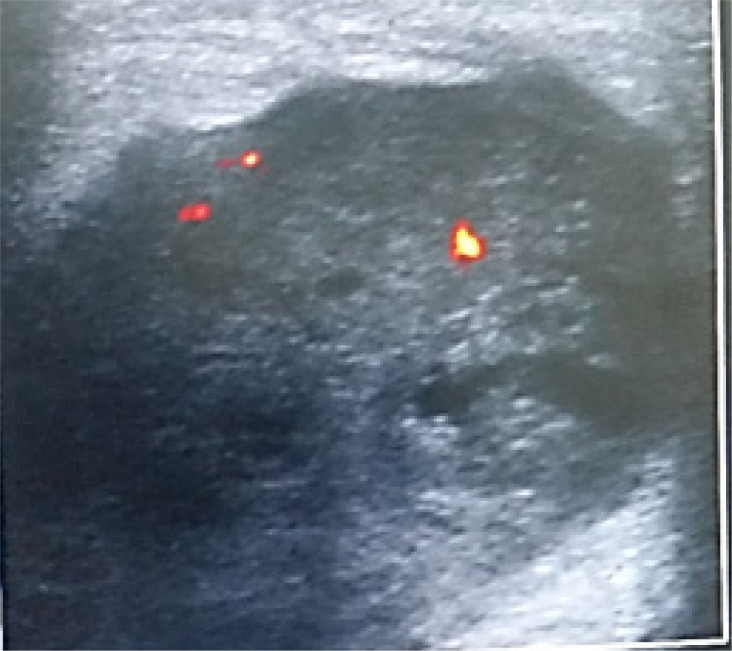
Complex cyst (type 4) > 50% solid with cystic foci A 55-year-old female with a hard non-tender left breast mass Ultrasound scan showing a predominantly solid mass which is vascular, with cystic spaces and angular margins Diagnosis: Invasive ductal carcinoma non-otherwise specified

The differential diagnoses of simple breast cysts were: simple epithelial cysts (Figure 2), fat necrosis, galactocele; The complicated cysts included galactocele (Figure 3), breast abscess, hematoma, fibrocystic disease, epidermoid cyst, post-operative seroma, invasive ductal carcinoma and the differential diagnoses of complex cysts were breast abscess, galactocele, fat necrosis, complicated (cystic) fibroadenoma, phyllodes tumor, intraductal papilloma, invasive papillary carcinoma and invasive ductal carcinoma (Figure 4).

## Discussion

The role of hormones and peptide are implicated in the development of breast cysts. Hence from our study, the highest incidence of breast cysts occurred among 20-29 years and 30-39 years age groups which is believed to be the peak of hormone function in women. Although our modal age is slightly lower than 30-50 years age group reported by Hines et al^1^ and Prassanti et al. 6 we still conform with previous reports 1,6,11-13 that breast cyst is mostly a disease of pre-menopausal women which may fluctuate in size and number according to the phase of the menstrual cycle. It diminishes in frequency after menopause therefore breast cysts in post-menopausal women should be treated with suspicion.

Breast diseases are not common in men thus accounting for the low male incidence (1.5%) of breast cysts in our study. Among the four male patients, complicated cyst was seen in one patient which had a benign histology. Out of the remaining three complex cysts, one had a benign histology while the other two were malignant.

The commonest presentation among our patients was a palpable lump. However, a few patients were asymptomatic, and the cysts were discovered during routine breast ultrasound.

Ultrasound is a very useful tool in discerning the details of cystic of breast masses. Using high-frequency transducers, small cysts 2mm to 3 mm in size can be detected and characterized. Spatial compounding helps to improve margin definition while tissue harmonics help to reduce noise.^2^,^3^ On doppler studies, internal vascularity should not be present in simple cysts, except there is a solid component which would warrant biopsy. Sonographically. a cyst can be evaluated for its shape, orientation, margin, wall thickness, internal features (echogenicity, septa, mass or solid component), posterior acoustic features, surrounding tissue vascularity. Based on these sonographic criteria, we identified: simple, complicated and complex cysts.

Simple cysts were typically benign in etiology and they constituted 33%of our cohort They were assigned BIRADS category 2 (Benign) and recommended for routine screening protocol by mammography. We did not suggest any further treatment for most of the patients except for those that underwent cyst aspiration on account of pain. The final histology was concordant with sonographic findings of benignity in simple cysts in 98.9% of the cases except for one patient (1.1%) in which malignancy was recorded. However, in the study by with Berg et al, 9 there was no malignancy recorded in simple cysts. The differential diagnosis for simple cysts included: simple epithelial cysts, early stages of hematoma, galactocele and fat necrosis as these lesions evolve in sonographic appearances over time.

Complicated cysts were seen in 22.6% of patients who could have been described ordinarily as having simple cysts, but for the presence of debris or thin septa. These internal echoes represent cellular debris, made up of proteins, blood products, and epithelial cells. Among the participants in the ACRIN trial,^14^ 14.1% had complicated cysts, which is lower than in our cohort. Because complicated cysts are not typically simple and for the fact that there exists a small (<2%) possibility of malignancy,^5^, ^7^ they were assigned BIRADS 3(Probably benign). From our study, among the 61 patients with complicated cysts, 96.7% were benign while 3.3% were malignant which is higher than previous reports.^15^-^18^ In a study by Venta et al,^15^ the malignancy rate of lesions classified as complicated cysts was 0.3%, Daly et al^16^ reported 0.44%. On the other hand, Kolbe et al^17^ and Chang et al^18^ span style=“font-family: ‘Times New Roman’”>did not report any malignancies among the complicated cysts in their study. Sometimes, a complicated cyst may be difficult to distinguish from a solid mass especially if it contains homogenous debris. In previous reports, 12% out of 517 lesions which were prospectively classified as complicated cysts proved to be solid lesions.^15^,^19^, The differential diagnosis of complicated cysts in our cohort were found to over-lap with simple cyst and they included hematoma, galactocele, fat necrosis/oil cyst. Others were breast abscess, fibrocystic disease, epidermoid cyst. We found that galactocele was the commonest pathology presenting as complicated cysts. It accounted for 21(34.4%) followed by abscesses 19(31.1%). However, Chang et al^18^ reported that abscesses constituted 60% of lesions among the complicated cysts.

We recorded 120 complex cysts, fulfilling the Berg criteria, making it the commonest type of breast cysts in this study. They accounted for 44.4% of all 270 cases. This is unlike other authors who reported that simple and complicated cysts predominated.^6^ ,^9^,^14^, ^20^

Complex cysts can be further classified according to presence of thick wall or septa (type1), presence of a mural nodule (type 2), predominantly cystic with solid components (type 3) and predominantly solid with cystic spaces (type 4). Although these cysts were complex in their sonographic appearance, their pathologic results include both benign and malignant conditions. From our study, the diagnosis was: breast abscess, galactocele, intraductal papilloma, cystic fibroadenoma, invasive ductal carcinoma and invasive papillary carcinoma. Among the 120 cases of complex cysts, 77(64%) were benign while 43(35.8%) were malignant. Berg et al^9^ reported 23% while Omori et al^21^ recorded 43%

Because complex cysts are associated with varying incidences of malignancy, they were assigned BIRADS categories 4 or 5 and biopsy was recommended. There were however, two patients (0.7%) whose lesions were already biopsy proven to be malignant prior to imaging and they were classified as BIRADS 6.

## Conclusion

Understanding the sonographic categories of breast cysts and the knowledge of the possible differential diagnoses is important in evaluating of patients with cysts. Although they are predominantly benign, malignancies also occur and should be sought for in the clinical history and examination. Routine screening protocol is recommended for BIRADS 2 cysts while short-term interval follow-up should be recommended for benign BIRADS 3 lesions. Pathological confirmation may be warranted in indeterminate cases and especially in patients with complex cysts (BIRADS 4 and 5) because it is the cyst category most frequently associated with breast cancer.
